# Characterization of Ageing- and Diet-Related Swine Models of Sarcopenia and Sarcopenic Obesity

**DOI:** 10.3390/ijms19030823

**Published:** 2018-03-12

**Authors:** Consolacion Garcia-Contreras, Marta Vazquez-Gomez, Laura Torres-Rovira, Jorge Gonzalez, Esteban Porrini, Magali Gonzalez-Colaço, Beatriz Isabel, Susana Astiz, Antonio Gonzalez-Bulnes

**Affiliations:** 1 Comparative Physiology Group, SGIT-INIA, 28040 Madrid, Spain; garcia.consolacion@inia.es (C.G.-C.); torres.laura@inia.es (L.T.-R.); astiz.susana@inia.es (S.A.); 2 Faculty of Veterinary Sciences, Universidad Complutense de Madrid, 28040 Madrid, Spain; mvgomez@ucm.es (M.V.-G.); bisabelr@ucm.es (B.I.); 3 Micros Veterinaria, Campus de Vegazana, 24007 Leon, Spain; info@microsvet.com; 4 Institute of Biomedical Technology (ITB), Universidad de La Laguna, 38200 Tenerife, Spain; esteban.l.porrini@gmail.com; 5 Central Unit of Clinical Research and Clinical Assays (UCICEC), Universitary Hospital of Canary Island, 28010 Tenerife, Spain; magaligch@hotmail.com

**Keywords:** animal-model, insulin-resistance, lipotoxicity, obesity, sarcopenia, swine

## Abstract

Sarcopenia and sarcopenic obesity are currently considered major global threats for health and well-being. However, there is a lack of adequate preclinical models for their study. The present trial evaluated the suitability of aged swine by determining changes in adiposity, fatty acids composition, antioxidant status and lipid peroxidation, development of metabolic disturbances and structural changes in tissues and organs. Iberian sows with clinical evidence of aging-related sarcopenia were fed a standard diet fulfilling their maintenance requirements or an obesogenic diet for 100 days. Aging and sarcopenia were related to increased lipid accumulation and cellular dysfunction at both adipose tissue and non-adipose ectopic tissues (liver and pancreas). Obesity concomitant to sarcopenia aggravates the condition by increasing visceral adiposity and causing dyslipidemia, insulin resistance and lipotoxicity in non-adipose tissues. These results support that the Iberian swine model represents certain features of sarcopenia and sarcopenic obesity in humans, paving the way for future research on physiopathology of these conditions and possible therapeutic targets.

## 1. Introduction

Sarcopenia is a syndrome characterized by loss of skeletal muscle mass and function [1], causing negative health outcomes like functional decline and disability [2,3,4] and increased mortality [5]. This may be further aggravated in case of obesity and increases in fat mass (sarcopenic obesity; [6]) which increases mobility and disability problems [7]. Currently, around 20 and 30% of elderly women and men, respectively, may have sarcopenic obesity [8]. Sarcopenic obesity is also related to metabolic disturbances, like metabolic syndrome and type-2 diabetes [9]; it is, therefore, considered a major global threat to health and well-being [10].

There are currently no drugs for the treatment of sarcopenia [7,11] and the recommended therapeutic interventions are based on lifestyle changes [9], with limited effects and with difficult implementation in elderly individuals with chronic diseases or different disabilities. Hence, there is a strong need for research efforts on pathogenesis and possible treatments. The results will contribute to relevant improvements of individual life-quality and savings in health-care resources.

Almost all the available studies are observational (with their inherent constraints), and interventional research is, therefore, necessary. Such research should be based on adequate translational animal models as elegantly reviewed by Palus in 2017 [12]. Most of the interventional trials on sarcopenia have been performed in rodents and have been based on induced acute muscle atrophy. These models do not represent sarcopenia by ageing [9], which needs long time to be achieved (around 20–24 months [13]). Moreover, the study of metabolic implications of sarcopenic obesity in rodents is constrained by their marked differences in metabolic and endocrine pathways with humans [14]. There is, consequently, a need for complementary preclinical animal models. The use of non-rodent mammals is essential to develop more relevant and predictive models of human disease. In this way, large animals have close similarities to humans in size and in many anatomical, physiological and pathological features. Moreover, large animal models of human diseases can be studied employing the same clinical approach used for human patients. In this sense, the pig has more similar patho-physiological features to humans than rodents [15].

Pigs used for biomedical research can be either random- or purpose-bred. Pigs from random-bred are typically farm animals (e.g., Landrace, Large White, Pietrain), where genetic selection is focused on animal production parameters. Purpose-bred pigs for biomedical research originate from closed colonies selected for a specific phenotype. The most commonly used swine breeds for research on obesity and metabolic diseases are Göttingen, Yucatan and Ossabaw Island pigs. However, all these breeds have a common ancestor: the Iberian pig.

The Iberian pig has an ancient origin, being traced back approximately to year 1000 Before Christ, and has been traditionally reared under extensive free-range conditions, using natural resources like pastures and acorns. Maintenance in semi-feral conditions has induced the pigs to develop an adaptive mechanism to the environment, which is known as *thrifty genotype* and which was firstly described in humans [16]. The *thrifty genotype* facilitates accommodation to seasonal cycles of feasting and famine because the ability to store fat in excess during food abundance enables survival during periods of scarcity. However, these individuals become obese in case of food-excess. Iberian pigs arise therefore as good model for studies on obesity and associated morbidities, since they develop cardiovascular and metabolic disorders in case of obesity, either during juvenile development [17] or adulthood [18].

The Iberian pig may be also useful for studies focused on sarcopenia, since the breed is reared under extensive traditional systems which facilitates the finding of older animals than in the case of breeds used for meat production under intensive management conditions (e.g., Landrace, Large-White, Pietrain). Intensive animal production systems are associated to intense genetic and productive selection and sows identified as having lower performance than the herd average are early culled, as early as age as 3–4 year-old [19]. Conversely, genetic and productive demands are not so strong in extensive traditional systems and culling is more related to either reproductive failures or musculoskeletal and locomotive disorders associated to age (which are similar to those associated with human sarcopenia).

Hence, the present study aimed to characterize a large-animal model for preclinical studies on sarcopenia and sarcopenic obesity, based on the use of old Iberian sows with aging-related sarcopenia and exposed or not to obesogenic diets. The validity of the models was assessed by determining possible links among adiposity, fatty acids composition, antioxidant status and lipid peroxidation, development of metabolic disturbances and structural changes in tissues and organs.

## 2. Results

### 2.1. Body-Weight and Adiposity

**Figure 1:**
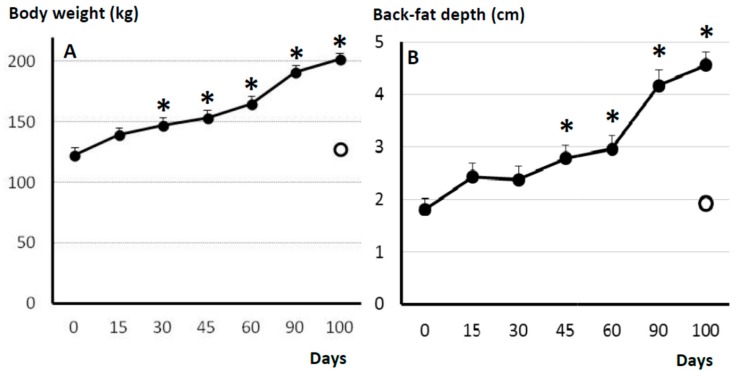
Mean (±S.E.M.) values in body-weight (**A**) and back-fat depth (**B**) in control sows (white dot; *n* = 17) and changes in such values over time after starting differential feeding with a diet enriched with saturated fat in treated sows (black line and dots; *n* = 11). Asterisks (*) denote significant differences between obese and non-obese sows (*p* < 0.05).

All the animals fed with the obesogenic diet showed a significant increase in body-weight (123 ± 6.1 to 202.3 ± 4.9 kg, *p* < 0.0001) and adiposity (16.1 ± 2.4 to 45.6 ± 2.9 mm of back-fat depth, *p* < 0.0001) from Day 0 to 100 (Figure 1). The values in control sows were similar to the initial values in obese sows (127.9 ± 4.0 kg and 19.3 ± 1.3 mm, respectively). The macroscopic findings during necropsy indicated a severe intraabdominal fattening in obese sows, with large amounts of mesenteric fat covering all viscerae and mesenteric tissues. However, intramuscular fat content was similar in obese and non-obese sows (12.9 ± 1.1% vs. 12.8 ± 0.7%).

### 2.2. Fatty Acid Composition of Subcutaneous and Visceral Fat and Muscle

The composition of fatty acids (FA) was similar at the outer and inner layers of subcutaneous fat (Supplementary Tables S1 and S2). Overall, when compared to non-obese sows, obese females showed a higher monounsaturated FA (MUFA) content (*p* < 0.05) and lower contents of polyunsaturated FA (PUFA), n-3 and n-6 PUFA (*p* < 0.005 for all). Visceral fat in obese sows also evidenced a higher MUFA content (*p* < 0.0001; Supplementary Table S3) and a lower content of PUFA, n-3 and n-6 PUFA (*p* < 0.0001 for all) than non-obese females. There were no differences at subcutaneous fat, but visceral fat of obese females showed higher ratios of MUFA/saturated FA (SFA) and C18:1/C18:0 (*p* < 0.05 for both).

The analysis of the fatty acid composition in the *longissimus dorsi* showed major effects at the polar lipid fraction (Supplementary Table S4). Obese sows showed a higher MUFA content and a lower content of SFA and PUFA than the controls and, as a consequence, higher MUFA/SFA and C18:1/C18:0 ratios (*p* < 0.005 for all). The content of n-3 and n-6 PUFA were lower, with a higher Σn-6/Σn-3 ratio, in obese than in control sows (*p* < 0.005 for all). The effects from obesity were minor at the neutral fraction (Supplementary Table S5), with MUFA content being lower in obese than in control females (*p* < 0.01).

### 2.3. Hepatic Architecture, Fatty Acid Composition and Function

**Figure 2:**
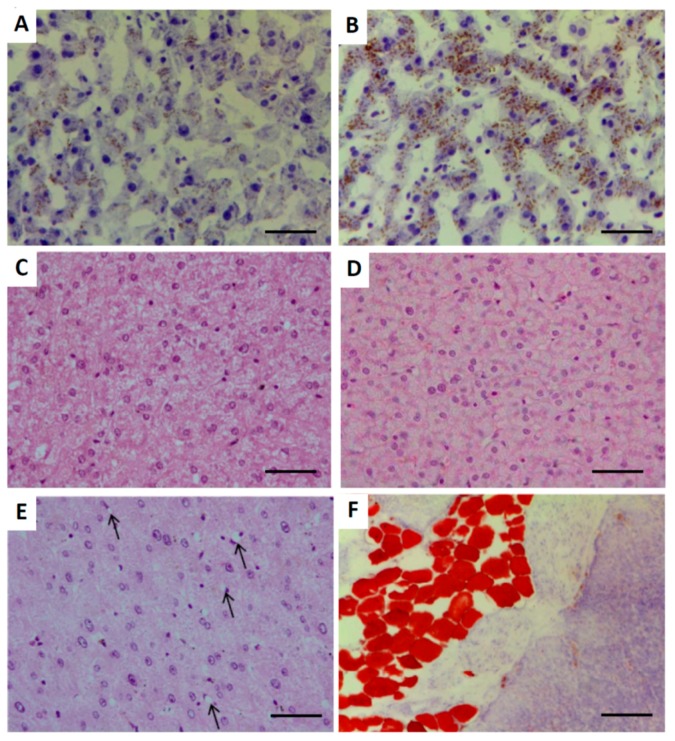
Histological images of liver and pancreas in obese sows. Upper pictures represent mild (**A**) and moderate (**B**) infiltration of lipids, stained in red, in the liver (oil red-O, 400×) Bar 50 µm. Pictures (**C**) and (**D**) of the liver represent mild and severe hydropic degeneration, respectively, whilst picture **E** exemplifies increased presence of lipocytes or Ito cells indicated with arrows (hematoxylin-eosin HE, 400×) Bar 50 µm. Picture **F** represents infiltration of adipocytes with intracytoplasmic accumulation of lipids, stained in red, in the pancreas (oil red-O, 100×) Bar 200 µm.

The total fat content in the liver was similar in control and obese groups (15.3 ± 0.2% vs. 14.5 ± 0.4%). Histological evaluation of the liver (Figure 2) indicated lipid accumulation in all the obese and in 82.4% of the control sows, with degrees varying from mild (63.6% and 76.5%, respectively) to moderate (36.4% and 23.5%, respectively). Increased presence of lipocytes (also known as perisinusoidal fat-storing cells, stellate cells or Ito cells) was found in all the obese and in 88.2% of the control sows. There were no evidences of inflammation or fibrosis, but hydropic degeneration was observed in 23.5% of the controls and all the obese animals (*p* < 0.05). All the controls had mild degeneration, whilst the obese sows showed mild (27.3%), moderate (63.7%, *p* < 0.05) or even severe degeneration (9.0%, *p* < 0.05).

The major effects of obesity on fatty acids composition of the liver, like in the muscle, were found at the polar fraction (Supplementary Table S6), with higher MUFA/SFA, C18:1/C18:0 and Σn-6/Σn-3 ratios in the obese than in the control females (*p* < 0.0001 for all). The neutral fraction (Supplementary Table S7) was characterized by a higher SFA content (*p* < 0.01) and a higher Σn-6/Σn-3 ratio (*p* < 0.0001) in the obese animals.

The obese sows showed no major changes in the plasma biomarkers of liver function throughout the study, excepting a significant increase in the concentrations of alkaline-phosphatase (56.5 ± 8.7 to 76.8 ± 10.1 IU/L, *p* < 0.05) and leucine-aminopeptidase (16.5 ± 1.9 to 21.4 ± 0.7 IU/L, *p* < 0.01). Conversely, albumin concentration decreased over time (4.6 ± 0.1 to 4.1 ± 0.1 g/dL; *p* < 0.05), from initial values similar to controls (4.7 ± 0.1 g/dL).

### 2.4. Plasma Lipid Profile

**Figure 3:**
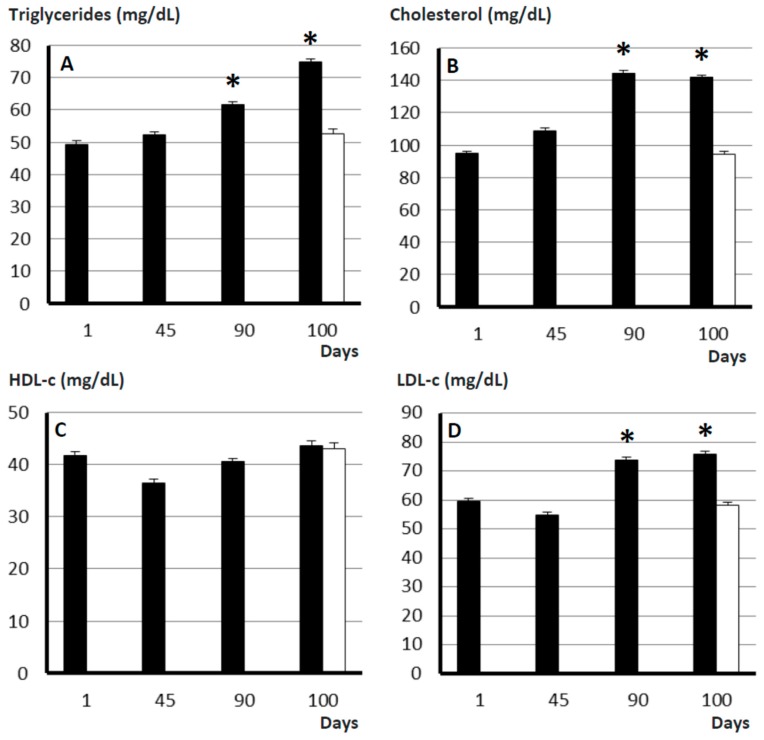
Mean (±S.E.M.) plasma concentrations of triglycerides (**A**), cholesterol (**B**), HDL-c (**C**) and LDL-c (**D**) in control sows (white bar; *n* = 17) and changes in such values over time after starting differential feeding with a diet enriched with saturated fat in treated sows (black bars; *n* = 11). Asterisks (*) denote significant differences between obese and non-obese sows (*p* < 0.05).

Plasma concentrations of triglycerides and cholesterol increased with time of treatment in the obese group (*p* < 0.05; Figure 3), starting from values similar to those of control sows at Day 0. The increase in total cholesterol was accompanied by a similar increase in low-density lipoproteins cholesterol (LDL-c) levels (*p* < 0.01) but not in high-density lipoproteins cholesterol (HDL-c). Hence, total cholesterol/HDL-c, LDL-c/HDL-c and atherogenic dyslipidemia ratios increased with time (*p* < 0.05).

### 2.5. Plasma Antioxidant Capacity and Lipid Oxidation

The plasma antioxidant capacity (measured by FRAP; ferric reducing antioxidant power assay) decreased in the obesogenic-diet group (from 23.6 ± 2.7 at Day 0 to 16.9 ± 2.6 µmol/mL at Day 100, *p* < 0.05), starting from values similar to those of control animals (24.6 ± 4.0 µmol/mL). Concomitantly, the values for total lipid oxidation (assessed as MDA; malondialdehyde) increased with time of treatment in the obese group (from 10.7 ± 0.6 at Day 0 to 12.3 ± 0.3 µmol/mL at Day 100, *p* < 0.05), starting from values similar to those of controls (10.6 ± 0.4 µmol/mL).

### 2.6. Pancreatic Architecture and Exocrine Function

Microscopic evaluation of the pancreas showed increased lipid accumulation in all the obese sows. Around half of them showed severe infiltration (>66% of the cells; 45.5%), whilst the remaining obese females showed moderate (34–66%; 27.3%) or mild infiltration (5–33%; 27.3%). Conversely, lipid accumulation was found in 12 of the 17 controls (70.6%, *p* < 0.05), with degrees varying from mild (47.1%) to moderate (23.5%), but not severe (*p* < 0.01 with obese sows). On the other hand, no inflammation, fibrosis or cellular injury were observed in any of the animals. Concomitantly, the assessment of plasma biomarkers of exocrine pancreatic function (lipase and amylase) showed no major changes during the period of study.

### 2.7. Pancreatic Endocrine Function and Insulin Resistance

**Figure 4:**
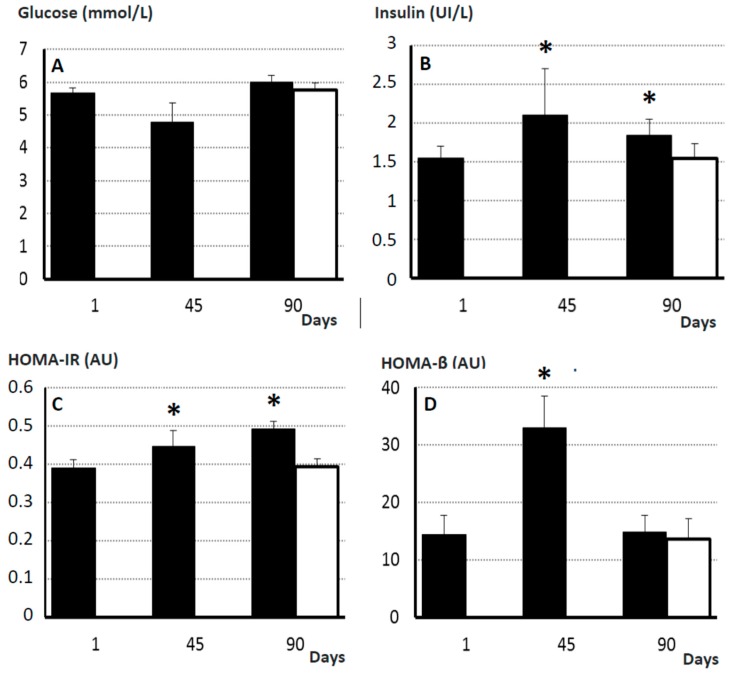
Mean (±S.E.M.) plasma concentrations of glucose (**A**) and insulin (**B**) and values for HOMA-IR and HOMA-β indexes (**C** and **D**, respectively) in control sows (white bar; *n* = 17) and changes in such values over time after starting differential feeding with a diet enriched with saturated fat in treated sows (black bars; *n* = 11). Asterisks (*) denote significant differences between obese and non-obese sows (*p* < 0.05).

**Figure 5:**
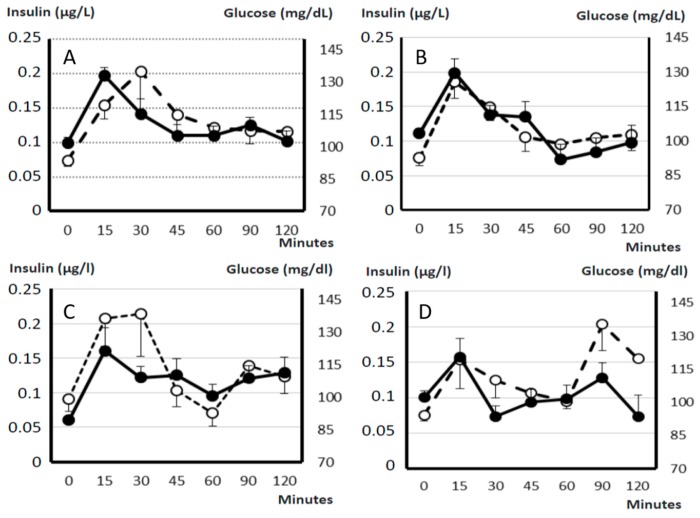
Changes in plasma concentration of glucose (continuous line with black circles) and insulin (discontinuous line with white circles) over time after oral administration of 2 g/kg live weight of d-glucose in control sows (**A**; *n* = 17) and at 0 (**B**), 45 (**C**) and 90 days (**D**) after starting differential feeding with a diet enriched with saturated fat in treated sows (*n* = 11). Areas under the Curve (AUCs) for glucose and insulin were similar in control and treated sows at Day 0. AUCs for glucose remained almost stable at 45 and 90 days but AUCs for insulin increased and were higher at both Day 45 and 90 than at Day 0 (*p* < 0.01 and *p* < 0.05, respectively).

Mean plasma concentrations of glucose and insulin, and HOMA-IR (Homeostatic Model Assessment for Insulin Resistance) and HOMA-β (Homeostatic Model Assessment for β-cell function) values were similar in controls and obese sows at Day 0. Afterwards, plasma glucose concentrations in obese females were maintained by changes in insulin secretion throughout the period of study (Figure 4). Insulin and HOMA-β index increased at Day 45 (*p* < 0.05 and *p* < 0.01, respectively) and decreased again at Day 90 (*p* < 0.05 and *p* < 0.01, respectively). On the other hand, HOMA-IR increased throughout the study (*p* < 0.05), evidencing insulin resistance (IR). Changes in insulin secretion and glucose elimination were confirmed when analyzing the OGTTs (Oral Glucose Tolerance Tests) performed during the study (Figure 5). In this way, the areas under the curve (AUCs) for glucose and insulin were similar in control and treated sows at Day 0. Afterwards, at 45 and 90 days, AUCs for glucose remained almost stable whilst AUCs for insulin increased 55.7% at Day 45 and 28.3% at Day 90 when compared to Day 0 (*p* < 0.01 and *p* < 0.05, respectively).

## 3. Discussion

The present study characterizes aged Iberian pigs as a robust, amenable, and reliable translational model for studies on sarcopenia and sarcopenic obesity. The model represents certain characteristics of the human disease, since aging and sarcopenia were related to increased lipids accumulation and cellular dysfunction, not only at the adipose tissue but also at non-adipose ectopic tissues (liver and pancreas). The food-intake excess leading to sarcopenic obesity worsened the condition by increasing visceral adiposity, dyslipidemia, insulin resistance and lipotoxicity in non-adipose tissues.

### 3.1. Similarities of the Model with Human Sarcopenia

In the pigs of the current study, aging was characterized, like in humans, by a significant reduction in muscle mass; even 50% when compared to values obtained in younger adults. This sarcopenic state was related to systemic dyslipidemia, insulin resistance (IR) and lipotoxicity in adipose tissue, liver and pancreas (i.e., increased lipids accumulation and cellular dysfunction).

The comparison of lipid profiles in the current trial with data obtained in younger adult pigs in previous studies [18] shows that plasma concentrations of triglycerides and total- and LDL-cholesterol are significantly higher in aged than in young adult sows (around 50 vs. 25 mg/dL for triglycerides, around 95 vs. 55 mg/dL for total cholesterol and around 55 vs. 25 mg/dL for LDL-c). Conversely, increases in HDL-c were smaller (around 35 vs. 25 mg/dL). These data indicate that in our model, ageing and sarcopenia induce a dyslipidemic profile similar to that described in humans [20], resembling the so-called “lipid triad” (elevated triglycerides and LDL-c with normal HDL-c; [21]). In consequence, aged sows have an increased atherogenic dyslipidemia ratio evidencing cardiometabolic risk like human beings [22].

In human medicine, elevated triglycerides in blood and tissues have been largely linked to establishment of glucose intolerance and IR [23,24] and there is a well-known connection between aging, sarcopenia and IR [20]. Concomitantly, in our sows, assessment of the glycemic index showed that HOMA-IR and HOMA-β indexes are higher in aged and sarcopenic individuals than in younger individuals.

The states of dyslipidemia and IR were accompanied by histological evidences of lipotoxicity in the liver, with morphological alterations similar to those found in the early nonalcoholic fatty liver disease (NAFLD) in humans. This finding indicates the main roles of IR and abnormal lipoprotein metabolism in the pathogenesis of NAFLD in swine, like in humans [25]. Around 80–90% of the non-obese sarcopenic sows evidenced lipids infiltration and increased presence of lipocytes (mostly known as Ito cells). This finding supports the relationship between ageing and proliferation of Ito cells previously described in human patients [26]. In 24% of our sows, the presence of ballooning injury, if extrapolated to humans, would be indicative of predisposition to a later development of nonalcoholic steatohepatitis (NASH) [25,27], a condition also known as lipotoxic liver disease [28]. The primary role of Ito cells is the intracytoplasmic storage of fat and vitamin A [29]. However, damage of the liver tissue is associated with proliferation of Ito cells, which plays an important role in the pathogenesis of chronic liver disease [30] since the cells are subsequently transformed in myofibroblasts. Such a process results in fibrogenesis and, in consequence, the normal hepatic tissue is replaced by fibrotic tissue in advanced stages. There was no evidence of hepatic fibrosis in the current study, which indicates early stages of liver disease. This assumption is reinforced by the lack of significant changes in most of the enzymes used as markers of hepatic function that remained within physiological ranges during the whole study [31,32].

Hence, the state of sarcopenia induced by aging in the Iberian sows of the present study represents certain clinical and histological evidences of dyslipidemia, IR, lipotoxicity, NAFLD, and ultimately prodrome of NASH, in human medicine.

### 3.2. Similarities of the Model with Human Sarcopenic Obesity

The obesogenic diet offered to sarcopenic sows affected body weight and adiposity very early after starting the differentiated feeding, like previously described for younger pigs [18].

In the obese sows of the current study, increases in fat accretion were mainly observed at subcutaneous and visceral compartments. In human medicine, it is well-known that aging-related increases in visceral fat favor the development of dyslipidemia and IR [33]. In our model, increases in visceral fat were similarly related to significant changes in the plasma lipids profile, which supports that the lipidomic profile of swine is mainly determined by the visceral fat, as found in humans [33]. The increase in total cholesterol and LDL-c (indicating hyperlipidemia type-2) concurrently with maintained low HDL-c levels found in our obese sarcopenic sows, if extrapolated to human medicine, would be considered as indicative of type 2 of diabetes mellitus (T2DM; [34]. In our pigs, such diagnosis was confirmed by the evidence of impaired glucose regulation throughout the study, with altered β-cell function and IR. However, there were no changes in plasma glucose concentrations, like in the first stages of the human disease [35], and our sows were able to counterbalance the prodrome of T2DM at Day 90. These findings may be related to the short duration of the experiment and/or to the high plasticity of the swine pancreas, in agreement with previous studies [36]. Hence, the data obtained so far pave the way for further studies assessing IR in longer experimental trials.

An elegant revision of Mlinar and Marc [37] analyzes the different factors and steps in the link between adiposity and IR. In brief, a triglyceride overload in non-adipose ectopic tissues occurs when the capacity of the adipose tissue for storing lipids is exceeded. At the same time, the failure of the vasculature to expand together with the adipocyte hypertrophy causes hypoxia of the adipose tissue and, therefore, oxidative stress; in turn, oxidative stress is reinforced by an altered metabolism of the non-adipose tissue. Systemic oxidative stress and steatosis in non-adipose tissues induce low-grade inflammation. Finally, the concurrence of hypertriglyceridemia and ectopic lipid deposition, increased oxidative stress and inflammation trigger IR. All these factors described in humans were also found in the obese sarcopenic sows of the current study.

Moreover, in our study, the assessment of fatty acid composition of subcutaneous and visceral fat, where more than 95% were neutral lipids (triglycerides), offers outstanding information on its changes during sarcopenic obesity. In brief, obesity increases MUFA content and decreases PUFA content (specifically n-3 and n-6 PUFA) at both compartments, but also increases the ratios of MUFA/SFA and C18:1/C18:0 in visceral fat (i.e., increases desaturation index and Stearoyl-CoA desaturase 1 activity; SCD1). In humans, increased desaturation index and SCD1 activity have been related to metabolic disorders, like alterations in lipogenesis and insulin regulation [23,38]. Similar results have been found in the Iberian pig in previous studies [39,40].

In the present study, we observed major effects of obesity on fatty acid composition of the polar fraction of cell membranes at non-adipose ectopic tissues (muscle and liver). Specifically, obese sows showed higher MUFA/SFA, C18:1/C18:0 and Σn-6/Σn-3 ratios than control sows. The implications of the two first ratios have been previously considered, but the changes in the Σn-6/Σn-3ratio also indicate important metabolic impairments. In the skeletal muscle, the n-3 PUFA content of cellular membranes plays a main role favoring the action of insulin; a high Σn-6/Σn-3ratio seems to be deleterious to insulin sensitivity [41] and ultimately results in IR [42]. In the liver, the increase of Σn-6/Σn-3ratio indicates also a pro-inflammatory state related to increased peripheral lipolysis and, therefore, enlarged flux of fatty acids [43].

The histological evidence of lipotoxicity at the liver previously described in a high percentage of the non-obese sarcopenic sows of the current study were also observed in the obese sows. All of them (100%) evidenced lipid infiltration, increased presence of Ito cells and presence of ballooning injury indicating nonalcoholic fatty liver disease (NAFLD) and later development of NASH. These data again mimic results found in aged humans. The increasing prevalence of NAFLD with age was highlighted in the Rotterdam study [44] while its relationship with sarcopenia has been reported, two years ago, in the Korean Sarcopenic Obesity Study [45]. Further analysis of this last database highlighted that this association is independent of obesity [46]. However, these studies are precluded by the inherent limitation in conducting invasive experimentation in humans; hence, animal models for NAFLD are necessary to better understand this pathogenesis. A recent comprehensive review of studies with rodent models of NASH [47] states that the ideal model should encompass all the defining features of the human condition (obesity, IR, steatohepatitis, and ultimately fibrosis). However, no single murine model currently represents all subsets of human NASH. In this scenario, swine models have an outstanding translational value.

In humans, the fatty liver is also closely associated with increased pancreatic fat content (a disorder also known as nonalcoholic fatty pancreas disease, NAFPD) [48]. NAFPD is associated with visceral obesity, dyslipidemia, IR and ultimately T2DM [49,50], which is similar to data from swine models [51]. A similar relationship was found in our current study, where pancreatic steatosis was severe in half of the obese sows and moderate or mild in the remaining animals; conversely, only 70% of the controls showed steatosis and most of them showed it to a mild degree. Moreover, all the obese sows showed pancreatic lipomatosis, defined as fat accumulation around and within the pancreas. This is a condition associated with aging and IR and known to worsen pancreatic dysfunction caused by steatosis [52]. Hence, these results also reinforce the translational value of our model for studies on the effect of aging, obesity and IR on the development of NAFPD.

## 4. Materials and Methods

### 4.1. Ethics Statement

The experiment was performed in agreement with the Spanish Policy for Animal Protection RD1201/05, which meets the European Union Directive 86/609. The experiment was specifically assessed and approved by the Committee of Ethics in Animal Research of the National Institute of Agricultural and Food Research and Technology (INIA) (report CEEA 2012/012, 28 January 2012). The sows were housed at the animal facilities of INIA, which meet local, national and European requirements.

### 4.2. Animals and Management

The experiment involved 28 Iberian sows (8–10 years-old) with established sarcopenia. Presence of sarcopenia was assessed by ultrasonographic evaluation of muscle mass (cross-sectional diameter of the *longissimus dorsi*), with a 5–8 MHz lineal-array probe (SonoSite Inc., Bothell, WA, USA), since ultrasonography is accepted as a reliable method for evaluating muscle mass and sarcopenia [53]. Muscle diameter had a mean value of 21.9 ± 3.2 mm, which is 50% lower than values obtained in adults (2 years-old; around 38–40 mm) and similar to data from young individuals (6 months-old; around 20 mm) of the same breed reared at our facilities.

All the sows were fed, prior to the experimental procedure, with a standard diet (2.8% of polyunsaturated fat and 3.08 Mcal/kg of metabolizable energy) fulfilling their maintenance requirements (2.5 kg/animal/day). For 100 days, seventeen sows (control group) continued being fed with the same diet and amount whilst 11 sows randomly chosen (obese group) were fed with an obesogenic diet (6.8% of saturated fat and 3.36 Mcal/kg of metabolizable energy) for inducing obesity [18]. Meal intake in the obese group was 4 kg/animal/day for the first 45 days and 5 kg/animal/day for the following 55 days.

### 4.3. Evaluation of Body Weight and Subcutaneous Adiposity

Changes in body-weight and subcutaneous fat-depth in the obese group were measured every 15 days, from Day 0 to 90, and finally at Day 100 together with the control group. Fat depth was evaluated with the ultrasound probe previously described, at the right-side at 4 cm from the midline and the head of the last rib.

### 4.4. Blood and Tissue Sampling

Plasma samples for assessment of glucose and lipids metabolism were drawn, after fasting, at Days 0, 45, 90 and 100 in the obese group and at Day 100 in the control group and immediately biobanked at −80 °C. Samples obtained at Days 0 and 100 in the obese group and at Day 100 in the control group were also used to determine redox status. In addition, oral glucose tolerance tests (OGTTs) were performed at Days 0, 45 and 90 in the obese and at Day 90 in the control sows by serial blood samplings (0, 15, 30, 60, 90 and 120 min) after giving 2 g/kg live-weight of d-glucose by gavage through a gastric tube.

At the end of the study (Day 100), a systematic necropsy was performed in all the animals, assessing the macroscopic appearance of the organs. Immediately, two portions of subcutaneous and visceral (perirenal) fat, muscle (*longissimus dorsi*), pancreas and left lobe of the liver were collected in 2-mL cryotubes and biobanked at −80 °C for assessment of fatty acids and/or oil red-O staining. A third portion of each tissue was fixed in 10% neutral-buffered-formalin and processed for hematoxylin-eosin (HE).

### 4.5. Assessment of Plasma Lipid Profiles

Plasma concentrations of triglycerides, total cholesterol and high-density and low-density lipoproteins cholesterol (HDL-c and LDL-c, respectively) were measured with a clinical chemistry analyzer (Saturno 300-plus; Crony Instruments s.r.l., Rome, Italy). Plasma HDL-c ratio and LDL-c ratio were calculated by dividing total cholesterol by HDL-c and LDL-c concentrations, respectively; plasma LDL-c/HDL-c ratio was obtained by dividing LDL-c by HDL-c concentrations. Finally, the atherogenic dyslipidemia ratio was calculated as log(triglycerides)/HDL-c.

### 4.6. Assessment of Plasma Antioxidant Capacity and Lipid Peroxidation

Values for total antioxidant capacity were determined by FRAP (ferric reducing antioxidant power assay) [54] whilst lipids oxidative damage was assessed by MDA (malondialdehyde) [55].

### 4.7. Assessment of Hepatic and Exocrine Pancreatic Function

Plasma concentrations of alanine-transaminase, albumin, alkaline-phosphatase, amylase, aspartate-aminotransferase, total and direct bilirubin, creatinine, γ-glutamyl-transpeptidase, leucine-aminopeptidase, lipase and urea were measured with the Saturno 300-plus analyzer (Crony Instruments s.r.l., Rome, Italy).

### 4.8. Assessment of Glucose Metabolism and Endocrine Pancreatic Function

Plasma concentrations of glucose and insulin were measured, respectively, with the Saturno 300-plus analyzer and with a Porcine Insulin ELISA kit (Mercodia AB, Uppsala, Sweden; 0.26 IU/L of assay sensitivity and 3.5% of intra-assay variation coefficient). Insulin sensitivity/resistance were determined, concomitantly with data from OGTTs, throughout the study of the HOMA-IR index [(FINS × FGLU)/22.5], whilst possible changes in β-cell function were assessed by the HOMA-β index [(20 × FINS)/(FGLU − 3.5)]. FINS accounts for fasting plasma insulin concentration in U/L and FGLU for fasting plasma glucose in mmol/L.

### 4.9. Histological Assessment of Liver and Pancreas

The stains of liver and pancreas tissue were examined by light microscopy and blindly scored for the presence and severity of histological features indicating fat infiltration, steatosis, inflammation, ballooning injury, fibrosis and vacuolization. Evaluation was based on the scoring developed by Kleiner et al. [56], considering none (<5%), mild (5–33%), moderate (34–66%) and severe degree (>66%).

### 4.10. Evaluation of Fatty Acids Composition

Fatty acid composition was analysed in subcutaneous (after differentiation of outer and inner layers) and visceral fat, *longissimus dorsi* muscle and liver. Fat was extracted using the *Ball-mill procedure* [57] and gas-chromatography was used for identification and quantification of fatty acids (FA) in total lipid extracts at subcutaneous and visceral fat, and separately for neutral (triacylglycerols or triglycerides) and polar (phospolipids) lipids at muscle and liver [58]. Total saturated FA (SFA), monounsaturated FA (MUFA) and polyunsaturated FA (PUFA) and total content of n-3 and n-6 PUFA and their ratio (Σn-6/Σn-3) were calculated from individual FA percentages. Finally, the desaturation index was obtained from the ratio MUFA/SFA and the activity of the stearoyl-CoA desaturase enzyme 1 (SCD1) was inferred from the proportions of oleic and stearic acids (C18:1n-9 and C18:0; product and precursor of SCD1 activity, respectively).

### 4.11. Statistical Analysis

Effects of diet on body-weight, adiposity, fatty-acid composition, metabolic and antioxidant/oxidative status were assessed by ANOVA for repeated measures (split-plot ANOVA), whilst changes over time were determined by Pearson correlation procedures. Histological features were assessed by one-way ANOVA or by a Kruskall–Wallis test if a Levene’s test showed non-homogeneous variables; Duncan’s post-hoc test was performed to contrast the differences among groups. Statistical analysis of results expressed as percentages was performed after arc-sine transformation of the values for each individual percentage, while the response to OGTTs was compared after calculating the Area under the Curve (AUC). All results were expressed as mean ± SEM and statistical significance was accepted from *p* < 0.05.

## 5. Conclusions

The present study shows that the Iberian swine model represents features of sarcopenia and sarcopenic obesity in humans, paving the way for future research on physiopathology of the condition and possible therapeutic targets. The strength of the model is increased by the availability and low-price of aged individuals of this breed and the short time necessary for inducing obesity. The present study was performed on female individuals, since availability of aged sows is higher than aged boars and because management is easier in females, but sex-related effects may be studied on males in further studies.

## Supplementary Materials

Supplementary materials can be found at http://www.mdpi.com/1422-0067/19/3/823/s1.
