# Supplementary material for: Characterization of Ageing- and Diet-Related Swine Models of Sarcopenia and Sarcopenic Obesity
Source: Int J Mol Sci. 2018 Mar 12;19(3):823. doi: 10.3390/ijms19030823 (PMC5877684; doi:10.3390/ijms19030823)
Supplement: Supplementary file 1 [file ijms-19-00823-s001.zip › TABLA 1.docx]

|  |  | | CONTROL | |  | OBESE | |  |  |
| --- | --- | --- | --- | --- | --- | --- | --- | --- | --- |
| **Trivial name** | **Abbreviation** | **Mean** | | **SEM** | | **Mean** | **SEM** | | **P-value** |
| **Myristic acid** | **C14:0** | 1.160 | | 0.032 | | 1.324 | 0.033 | | 0.002 |
| **Palmitic acid** | **C16:0** | 20.553 | | 0.421 | | 22.420 | 0.571 | | 0.013 |
| **cis-7 hexadecenoic acid** | **C16:1 n-9** | 0.494 | | 0.039 | | 0.302 | 0.021 | | 0.001 |
| **Palmitoleic acid** | **C16:1 n-7** | 2.447 | | 0.081 | | 2.545 | 0.068 | | 0.404 |
| **Margaric acid** | **C17:0** | 0.370 | | 0.012 | | 0.277 | 0.011 | | 0.000 |
| **cis-10-Heptadecenoic acid** | **C17:1** | 0.358 | | 0.011 | | 0.313 | 0.012 | | 0.012 |
| **Stearic acid** | **C18:0** | 9.853 | | 0.295 | | 9.697 | 0.415 | | 0.755 |
| **Oleic acid** | **C18:1 n-9** | 43.334 | | 0.638 | | 46.405 | 0.401 | | 0.001 |
| **cis-vaccenic acid** | **C18:1 n-7** | 2.887 | | 0.077 | | 2.801 | 0.092 | | 0.482 |
| **Linoleic acid** | **C18:2 n-6** | 13.212 | | 0.639 | | 9.854 | 0.484 | | 0.001 |
| **Linolenic acid** | **C18:3 n-3** | 1.027 | | 0.114 | | 0.679 | 0.052 | | 0.027 |
| **Eicosenoic acid** | **C20:1 n-9** | 2.026 | | 0.091 | | 1.746 | 0.063 | | 0.034 |
| **Mead acid** | **C20:3n-9** | 0.106 | | 0.006 | | 0.085 | 0.010 | | 0.057 |
| **Arachidonic acid** | **C20:4 n-6** | 1.207 | | 0.078 | | 0.877 | 0.072 | | 0.007 |
| **Eicosapentaenoic acid** | **C20:5 n-3** | 0.115 | | 0.005 | | 0.079 | 0.009 | | 0.001 |
| **Erucic acid** | **C22:1 n-9** | 0.127 | | 0.009 | | 0.104 | 0.004 | | 0.064 |
| **Adrenic acid** | **C22:4 n-6** | 0.400 | | 0.063 | | 0.267 | 0.046 | | 0.135 |
| **Docosapentaenoic acid** | **C22:5 n-3** | 0.206 | | 0.023 | | 0.148 | 0.033 | | 0.154 |
| **Docosahexaenoic acid** | **C22:6 n-3** | 0.117 | | 0.023 | | 0.077 | 0.014 | | 0.213 |
| **SFA^1^** |  | 11.383 | | 0.313 | | 11.299 | 0.442 | | 0.873 |
| **MUFA^2^** |  | 51.674 | | 0.659 | | 54.216 | 0.485 | | 0.010 |
| **PUFA^3^** |  | 16.390 | | 0.871 | | 12.065 | 0.650 | | 0.001 |
| **MUFA/SFA** |  | 4.598 | | 0.145 | | 4.886 | 0.225 | | 0.268 |
| **PUFAn-6^4^** |  | 14.819 | | 0.753 | | 10.997 | 0.592 | | 0.001 |
| **PUFAn-3^5^** |  | 1.464 | | 0.142 | | 0.983 | 0.068 | | 0.016 |
| **∑n-6/∑n-3** |  | 11.046 | | 0.698 | | 11.373 | 0.417 | | 0.730 |
| **C18:1/C18:0** |  | 4.760 | | 0.159 | | 5.183 | 0.256 | | 0.150 |

**Table 1. Fatty-acids composition.** Differences in mean values (%) and S.E.M. for total lipids in the outer layer of subcutaneous fat of control (normal diet) and obese sows (obesogenic diet).

^1^SFA = Saturated fatty acids; Includes: C14:0, C16:0, C17:0 and C18:0

^2^MUFA = Monounsaturated fatty acids; Includes: C16:1n-9, C16:1n-7, C17:1, C18:1n-9, C18:1n-7, C20:1n-9 and C22:1n-9.

^3^PUFA = Polyunsaturated fatty acids: Includes: C18:2n-6, C18:3n-3, C20:3n-9, C20:4n-6, C20:5n-3, C22:4n-6, C22:5n-3, C22:6n-3.

^4^Includes: C18.2n-6, C20:4n-6 and C22:4n-6.

^6^Includes: C18:3n-3, C20:5n-3, C22:5n-3 and C22:6n-3.
