# Supplementary material for: Characterization of Ageing- and Diet-Related Swine Models of Sarcopenia and Sarcopenic Obesity
Source: Int J Mol Sci. 2018 Mar 12;19(3):823. doi: 10.3390/ijms19030823 (PMC5877684; doi:10.3390/ijms19030823)
Supplement: Supplementary file 1 [file ijms-19-00823-s001.zip › TABLA 2.docx]

|  |  | | CONTROL | |  | OBESE | |  |  |
| --- | --- | --- | --- | --- | --- | --- | --- | --- | --- |
| **Trivial name** | **Abbreviation** | **Mean** | | **SEM** | | **Mean** | **SEM** | | **P-value** |
| **Myristic acid** | **C14:0** | 1.172 | | 0.033 | | 1.403 | 0.026 | | 0.000 |
| **Palmitic acid** | **C16:0** | 21.806 | | 0.374 | | 24.522 | 0.465 | | 0.000 |
| **cis-7 hexadecenoic acid** | **C16:1 n-9** | 0.514 | | 0.037 | | 0.304 | 0.020 | | 0.000 |
| **Palmitoleic acid** | **C16:1 n-7** | 2.061 | | 0.076 | | 2.314 | 0.060 | | 0.025 |
| **Margaric acid** | **C17:0** | 0.397 | | 0.014 | | 0.310 | 0.015 | | 0.000 |
| **cis-10-Heptadecenoic acid** | **C17:1** | 0.288 | | 0.028 | | 0.277 | 0.013 | | 0.770 |
| **Stearic acid** | **C18:0** | 11.880 | | 0.352 | | 12.003 | 0.408 | | 0.824 |
| **Oleic acid** | **C18:1 n-9** | 41.055 | | 0.619 | | 43.737 | 0.365 | | 0.003 |
| **cis-vaccenic acid** | **C18:1 n-7** | 2.481 | | 0.083 | | 2.408 | 0.089 | | 0.564 |
| **Linoleic acid** | **C18:2 n-6** | 13.496 | | 0.571 | | 9.269 | 0.382 | | 0.000 |
| **Linolenic acid** | **C18:3 n-3** | 1.079 | | 0.114 | | 0.691 | 0.062 | | 0.016 |
| **Eicosenoic acid** | **C20:1 n-9** | 1.754 | | 0.074 | | 1.434 | 0.044 | | 0.003 |
| **Mead acid** | **C20:3n-9** | 0.086 | | 0.009 | | 0.074 | 0.002 | | 0.320 |
| **Arachidonic acid** | **C20:4 n-6** | 1.021 | | 0.054 | | 0.664 | 0.042 | | 0.000 |
| **Eicosapentaenoic acid** | **C20:5 n-3** | 0.102 | | 0.011 | | 0.085 | 0.006 | | 0.254 |
| **Erucic acid** | **C22:1 n-9** | 0.150 | | 0.018 | | 0.114 | 0.005 | | 0.129 |
| **Adrenic acid** | **C22:4 n-6** | 0.314 | | 0.049 | | 0.196 | 0.034 | | 0.093 |
| **Docosapentaenoic acid** | **C22:5 n-3** | 0.216 | | 0.030 | | 0.111 | 0.015 | | 0.014 |
| **Docosahexaenoic acid** | **C22:6 n-3** | 0.131 | | 0.025 | | 0.084 | 0.007 | | 0.151 |
| **SFA^1^** |  | 13.448 | | 0.361 | | 13.715 | 0.420 | | 0.639 |
| **MUFA^2^** |  | 48.303 | | 0.624 | | 50.588 | 0.488 | | 0.014 |
| **PUFA^3^** |  | 16.443 | | 0.716 | | 11.175 | 0.512 | | 0.000 |
| **MUFA/SFA** |  | 3.639 | | 0.119 | | 3.732 | 0.145 | | 0.625 |
| **PUFAn-6^4^** |  | 14.830 | | 0.625 | | 10.130 | 0.448 | | 0.000 |
| **PUFAn-3^5^** |  | 1.527 | | 0.129 | | 0.971 | 0.068 | | 0.003 |
| **∑n-6/∑n-3** |  | 10.381 | | 0.574 | | 10.655 | 0.354 | | 0.726 |
| **C18:1/C18:0** |  | 3.721 | | 0.131 | | 3.898 | 0.160 | | 0.400 |

**Table 2. Fatty-acids composition.** Differences in mean values (%) and S.E.M. for total lipids in the inner layer of subcutaneous fat of control (normal diet) and obese sows (obesogenic diet).

^1^SFA = Saturated fatty acids; Includes: C14:0, C16:0, C17:0 and C18:0

^2^MUFA = Monounsaturated fatty acids; Includes: C16:1n-9, C16:1n-7, C17:1, C18:1n-9, C18:1n-7, C20:1n-9 and C22:1n-9.

^3^PUFA = Polyunsaturated fatty acids: Includes: C18:2n-6, C18:3n-3, C20:3n-9, C20:4n-6, C20:5n-3, C22:4n-6, C22:5n-3, C22:6n-3.

^4^Includes: C18.2n-6, C20:4n-6 and C22:4n-6.

^6^Includes: C18:3n-3, C20:5n-3, C22:5n-3 and C22:6n-3.
