# Supplementary material for: Characterization of Ageing- and Diet-Related Swine Models of Sarcopenia and Sarcopenic Obesity
Source: Int J Mol Sci. 2018 Mar 12;19(3):823. doi: 10.3390/ijms19030823 (PMC5877684; doi:10.3390/ijms19030823)
Supplement: Supplementary file 1 [file ijms-19-00823-s001.zip › TABLA 3.docx]

|  |  | | CONTROL | |  | OBESE | |  |  |
| --- | --- | --- | --- | --- | --- | --- | --- | --- | --- |
| **Trivial name** | **Abbreviation** | **Mean** | | **SEM** | | **Mean** | **SEM** | | **P-value** |
| **Myristic acid** | **C14:0** | 1.331 | | 0.050 | | 1.580 | 0.057 | | 0.004 |
| **Palmitic acid** | **C16:0** | 24.616 | | 0.476 | | 27.632 | 0.676 | | 0.001 |
| **cis-7 hexadecenoic acid** | **C16:1 n-9** | 0.746 | | 0.053 | | 0.365 | 0.041 | | 0.000 |
| **Palmitoleic acid** | **C16:1 n-7** | 1.593 | | 0.068 | | 2.277 | 0.079 | | 0.000 |
| **Margaric acid** | **C17:0** | 0.444 | | 0.059 | | 0.372 | 0.028 | | 0.361 |
| **cis-10-Heptadecenoic acid** | **C17:1** | 0.320 | | 0.012 | | 0.265 | 0.016 | | 0.012 |
| **Stearic acid** | **C18:0** | 15.150 | | 0.521 | | 14.974 | 0.606 | | 0.832 |
| **Oleic acid** | **C18:1 n-9** | 30.400 | | 0.527 | | 37.116 | 0.613 | | 0.000 |
| **cis-vaccenic acid** | **C18:1 n-7** | 1.600 | | 0.057 | | 1.858 | 0.051 | | 0.005 |
| **Linoleic acid** | **C18:2 n-6** | 19.823 | | 0.513 | | 10.800 | 0.762 | | 0.000 |
| **Linolenic acid** | **C18:3 n-3** | 1.352 | | 0.069 | | 0.756 | 0.081 | | 0.000 |
| **Eicosenoic acid** | **C20:1 n-9** | 0.844 | | 0.029 | | 0.934 | 0.031 | | 0.056 |
| **Mead acid** | **C20:3n-9** | -- | | -- | | -- | -- | | -- |
| **Arachidonic acid** | **C20:4 n-6** | 0.715 | | 0.031 | | 0.473 | 0.032 | | 0.000 |
| **Eicosapentaenoic acid** | **C20:5 n-3** | 0.119 | | 0.013 | | 0.079 | 0.006 | | 0.026 |
| **Erucic acid** | **C22:1 n-9** | 0.313 | | 0.023 | | 0.160 | 0.008 | | 0.000 |
| **Adrenic acid** | **C22:4 n-6** | 0.149 | | 0.015 | | 0.107 | 0.013 | | 0.062 |
| **Docosapentaenoic acid** | **C22:5 n-3** | 0.219 | | 0.017 | | 0.145 | 0.016 | | 0.007 |
| **Docosahexaenoic acid** | **C22:6 n-3** | 0.265 | | 0.024 | | 0.107 | 0.014 | | 0.000 |
| **SFA^1^** |  | 16.925 | | 0.579 | | 16.926 | 0.633 | | 0.999 |
| **MUFA^2^** |  | 35.816 | | 0.627 | | 42.975 | 0.675 | | 0.000 |
| **PUFA3** |  | 22.642 | | 0.619 | | 12.467 | 0.887 | | 0.000 |
| **MUFA/SFA** |  | 2.179 | | 0.120 | | 2.583 | 0.121 | | 0.034 |
| **PUFAn-6^4^** |  | 20.688 | | 0.544 | | 11.380 | 0.803 | | 0.000 |
| **PUFAn-3^5^** |  | 1.955 | | 0.095 | | 1.087 | 0.091 | | 0.000 |
| **∑n-6/∑n-3** |  | 10.827 | | 0.364 | | 10.597 | 0.394 | | 0.685 |
| **C18:1/C18:0** |  | 2.173 | | 0.117 | | 2.654 | 0.130 | | 0.013 |

**Table 3. Fatty-acids composition.** Differences in mean values (%) and S.E.M. for total lipids in visceral fat of control (normal diet) and obese sows (obesogenic diet).

^1^SFA = Saturated fatty acids; Includes: C14:0, C16:0, C17:0 and C18:0

^2^MUFA = Monounsaturated fatty acids; Includes: C16:1n-9, C16:1n-7, C17:1, C18:1n-9, C18:1n-7, C20:1n-9 and C22:1n-9.

^3^PUFA = Polyunsaturated fatty acids: Includes: C18:2n-6, C18:3n-3, C20:3n-9, C20:4n-6, C20:5n-3, C22:4n-6, C22:5n-3, C22:6n-3.

^4^Includes: C18.2n-6, C20:4n-6 and C22:4n-6.

^6^Includes: C18:3n-3, C20:5n-3, C22:5n-3 and C22:6n-3.
