# Supplementary material for: Characterization of Ageing- and Diet-Related Swine Models of Sarcopenia and Sarcopenic Obesity
Source: Int J Mol Sci. 2018 Mar 12;19(3):823. doi: 10.3390/ijms19030823 (PMC5877684; doi:10.3390/ijms19030823)
Supplement: Supplementary file 1 [file ijms-19-00823-s001.zip › TABLA 4.docx]

|  |  | | CONTROL | | |  | | OBESE | |  |  |
| --- | --- | --- | --- | --- | --- | --- | --- | --- | --- | --- | --- |
| **Trivial name** | **Abbreviation** | | **Mean** | | **SEM** | | | **Mean** | **SEM** | | **P-value** |
| **Myristic acid** | **C14:0** | | 3.505 | | 0.232 | | | 2.269 | 0.119 | | 0.001 |
| **Palmitic acid** | **C16:0** | 19.969 | | 0.165 | | | 21.443 | | 0.150 | | 0.000 |
| **cis-7 hexadecenoic acid** | **C16:1 n-9** | 0.711 | | 0.059 | | | 0.594 | | 0.033 | | 0.164 |
| **Palmitoleic acid** | **C16:1 n-7** | 0.649 | | 0.068 | | | 0.958 | | 0.026 | | 0.002 |
| **Margaric acid** | **C17:0** | 0.486 | | 0.012 | | | 0.473 | | 0.012 | | 0.473 |
| **cis-10-Heptadecenoic acid** | **C17:1** | 2.340 | | 0.198 | | | 3.656 | | 0.122 | | 0.000 |
| **Stearic acid** | **C18:0** | 8.597 | | 0.112 | | | 7.696 | | 0.166 | | 0.000 |
| **Oleic acid** | **C18:1 n-9** | 8.346 | | 0.221 | | | 10.260 | | 0.245 | | 0.000 |
| **cis-vaccenic acid** | **C18:1 n-7** | 4.235 | | 0.159 | | | 4.559 | | 0.115 | | 0.168 |
| **Linoleic acid** | **C18:2 n-6** | 30.755 | | 0.478 | | | 31.593 | | 0.395 | | 0.244 |
| **Linolenic acid** | **C18:3 n-3** | 0.520 | | 0.017 | | | 0.526 | | 0.014 | | 0.821 |
| **Eicosenoic acid** | **C20:1 n-9** | 0.273 | | 0.013 | | | 0.333 | | 0.010 | | 0.003 |
| **Mead acid** | **C20:3n-9** | 0.904 | | 0.039 | | | 0.572 | | 0.020 | | 0.000 |
| **Arachidonic acid** | **C20:4 n-6** | 15.625 | | 0.370 | | | 12.659 | | 0.198 | | 0.000 |
| **Eicosapentaenoic acid** | **C20:5 n-3** | 0.393 | | 0.042 | | | 0.076 | | 0.010 | | 0.000 |
| **Erucic acid** | **C22:1 n-9** | -- | | -- | | | -- | | -- | | -- |
| **Adrenic acid** | **C22:4 n-6** | 0.120 | | 0.007 | | | 0.239 | | 0.024 | | 0.000 |
| **Docosapentaenoic acid** | **C22:5 n-3** | 2.149 | | 0.091 | | | 1.704 | | 0.055 | | 0.002 |
| **Docosahexaenoic acid** | **C22:6 n-3** | 0.422 | | 0.036 | | | 0.391 | | 0.019 | | 0.538 |
| **SFA^1^** |  | 12.588 | | 0.249 | | | 10.438 | | 0.188 | | 0.000 |
| **MUFA^2^** |  | 16.553 | | 0.390 | | | 20.360 | | 0.307 | | 0.000 |
| **PUFA^3^** |  | 50.889 | | 0.300 | | | 47.759 | | 0.382 | | 0.000 |
| **MUFA/SFA** |  | 1.328 | | 0.050 | | | 1.956 | | 0.043 | | 0.000 |
| **PUFAn-6^4^** |  | 46.501 | | 0.288 | | | 44.490 | | 0.432 | | 0.001 |
| **PUFAn-3^5^** |  | 3.484 | | 0.134 | | | 2.697 | | 0.068 | | 0.000 |
| **∑n-6/∑n-3** |  | 13.677 | | 0.549 | | | 16.630 | | 0.548 | | 0.002 |
| **C18:1/C18:0** |  | 1.465 | | 0.031 | | | 1.936 | | 0.063 | | 0.000 |

**Table 4. Fatty-acids composition.** Differences in mean values (%) and S.E.M. for polar lipids in the *longissimus dorsi* of control (normal diet) and obese sows (obesogenic diet).

^1^SFA = Saturated fatty acids; Includes: C14:0, C16:0, C17:0 and C18:0

^2^MUFA = Monounsaturated fatty acids; Includes: C16:1n-9, C16:1n-7, C17:1, C18:1n-9, C18:1n-7, C20:1n-9 and C22:1n-9.

^3^PUFA = Polyunsaturated fatty acids: Includes: C18:2n-6, C18:3n-3, C20:3n-9, C20:4n-6, C20:5n-3, C22:4n-6, C22:5n-3, C22:6n-3.

^4^Includes: C18.2n-6, C20:4n-6 and C22:4n-6.

^6^Includes: C18:3n-3, C20:5n-3, C22:5n-3 and C22:6n-3.
