# Supplementary material for: Characterization of Ageing- and Diet-Related Swine Models of Sarcopenia and Sarcopenic Obesity
Source: Int J Mol Sci. 2018 Mar 12;19(3):823. doi: 10.3390/ijms19030823 (PMC5877684; doi:10.3390/ijms19030823)
Supplement: Supplementary file 1 [file ijms-19-00823-s001.zip › TABLA 5.docx]

**Table 5. Fatty-acids composition.** Differences in mean values (%) and S.E.M. for neutral lipids in the *longissimus dorsi* of control (normal diet) and obese sows (obesogenic diet).

|  |  | CONTROL | | OBESE | |  |
| --- | --- | --- | --- | --- | --- | --- |
| **Trivial name** | **Abbreviation** | **Mean** | **SEM** | **Mean** | **SEM** | **P-value** |
| **Myristic acid** | **C14:0** | 1.073 | 0.031 | 1.224 | 0.039 | 0.006 |
| **Palmitic acid** | **C16:0** | 20.620 | 0.277 | 22.531 | 0.589 | 0.003 |
| **cis-7 hexadecenoic acid** | **C16:1 n-9** | 0.617 | 0.031 | 0.377 | 0.029 | 0.000 |
| **Palmitoleic acid** | **C16:1 n-7** | 4.290 | 0.105 | 4.607 | 0.149 | 0.085 |
| **Margaric acid** | **C17:0** | 0.262 | 0.008 | 0.205 | 0.012 | 0.000 |
| **cis-10-Heptadecenoic acid** | **C17:1** | 0.322 | 0.010 | 0.315 | 0.032 | 0.827 |
| **Stearic acid** | **C18:0** | 8.950 | 0.313 | 9.312 | 0.443 | 0.499 |
| **Oleic acid** | **C18:1 n-9** | 48.345 | 0.339 | 46.346 | 0.625 | 0.005 |
| **cis-vaccenic acid** | **C18:1 n-7** | 4.263 | 0.155 | 4.443 | 0.133 | 0.424 |
| **Linoleic acid** | **C18:2 n-6** | 8.128 | 0.380 | 7.597 | 0.543 | 0.417 |
| **Linolenic acid** | **C18:3 n-3** | 0.672 | 0.067 | 0.528 | 0.088 | 0.197 |
| **Eicosenoic acid** | **C20:1 n-9** | 1.339 | 0.042 | 1.065 | 0.038 | 0.000 |
| **Mead acid** | **C20:3n-9** | 0.424 | 0.037 | 0.127 | 0.007 | 0.000 |
| **Arachidonic acid** | **C20:4 n-6** | 0.312 | 0.032 | 0.871 | 0.096 | 0.000 |
| **Eicosapentaenoic acid** | **C20:5 n-3** | 0.067 | 0.035 | 0.097 | 0.029 | 0.552 |
| **Erucic acid** | **C22:1 n-9** | 0.078 | 0.014 | 0.030 | 0.005 | 0.013 |
| **Adrenic acid** | **C22:4 n-6** | 0.083 | 0.008 | 0.104 | 0.017 | 0.216 |
| **Docosapentaenoic acid** | **C22:5 n-3** | 0.119 | 0.013 | 0.159 | 0.011 | 0.039 |
| **Docosahexaenoic acid** | **C22:6 n-3** | 0.037 | 0.008 | 0.061 | 0.012 | 0.110 |
| **SFA^1^** |  | 10.285 | 0.324 | 10.740 | 0.468 | 0.416 |
| **MUFA^2^** |  | 59.254 | 0.288 | 57.183 | 0.734 | 0.006 |
| **PUFA^3^** |  | 9.842 | 0.448 | 9.545 | 0.670 | 0.705 |
| **MUFA/SFA** |  | 5.859 | 0.198 | 5.449 | 0.288 | 0.236 |
| **PUFAn-6^4^** |  | 8.522 | 0.368 | 8.573 | 0.554 | 0.937 |
| **PUFAn-3^5^** |  | 0.896 | 0.076 | 0.845 | 0.121 | 0.715 |
| **∑n-6/∑n-3** |  | 10.215 | 0.555 | 11.304 | 0.894 | 0.283 |
| **C18:1/C18:0** |  | 5.998 | 0.218 | 5.607 | 0.324 | 0.308 |

^1^SFA = Saturated fatty acids; Includes: C14:0, C16:0, C17:0 and C18:0

^2^MUFA = Monounsaturated fatty acids; Includes: C16:1n-9, C16:1n-7, C17:1, C18:1n-9, C18:1n-7 and C22:1n-9.

^3^PUFA = Polyunsaturated fatty acids: Includes: C18:2n-6, C18:3n-3, C20:3n-9, C20:4n-6, C20:5n-3, C22:4n-6, C22:5n-3, C22:6n-3.

^4^Includes: C18.2n-6, C20:4n-6 and C22:4n-6.

^6^Includes: C18:3n-3, C20:5n-3, C22:5n-3 and C22:6n-3.
