# Supplementary material for: Characterization of Ageing- and Diet-Related Swine Models of Sarcopenia and Sarcopenic Obesity
Source: Int J Mol Sci. 2018 Mar 12;19(3):823. doi: 10.3390/ijms19030823 (PMC5877684; doi:10.3390/ijms19030823)
Supplement: Supplementary file 1 [file ijms-19-00823-s001.zip › TABLA 6.docx]

|  |  | | CONTROL | |  | OBESE | |  |  |
| --- | --- | --- | --- | --- | --- | --- | --- | --- | --- |
| **Trivial name** | **Abbreviation** | **Mean** | | **SEM** | | **Mean** | **SEM** | | **P-value** |
| **Myristic acid** | **C14:0** | 0.211 | | 0.009 | | 0.274 | 0.037 | | 0.060 |
| **Palmitic acid** | **C16:0** | 16.457 | | 0.448 | | 19.457 | 0.805 | | 0.002 |
| **cis-7 hexadecenoic acid** | **C16:1 n-9** | 0.293 | | 0.029 | | 0.579 | 0.032 | | 0.000 |
| **Palmitoleic acid** | **C16:1 n-7** | 0.479 | | 0.034 | | 0.614 | 0.150 | | 0.296 |
| **Margaric acid** | **C17:0** | 1.393 | | 0.074 | | 1.016 | 0.076 | | 0.002 |
| **cis-10-Heptadecenoic acid** | **C17:1** | 0.335 | | 0.023 | | 0.405 | 0.020 | | 0.043 |
| **Stearic acid** | **C18:0** | 28.521 | | 0.562 | | 28.969 | 0.846 | | 0.649 |
| **Oleic acid** | **C18:1 n-9** | 8.810 | | 0.234 | | 12.799 | 0.456 | | 0.000 |
| **cis-vaccenic acid** | **C18:1 n-7** | 1.213 | | 0.037 | | 1.392 | 0.052 | | 0.007 |
| **Linoleic acid** | **C18:2 n-6** | 15.404 | | 0.437 | | 16.644 | 0.432 | | 0.066 |
| **Linolenic acid** | **C18:3 n-3** | 0.348 | | 0.016 | | 0.308 | 0.029 | | 0.211 |
| **Eicosenoic acid** | **C20:1 n-9** | 0.324 | | 0.099 | | 0.418 | 0.060 | | 0.484 |
| **Mead acid** | **C20:3n-9** | -- | | -- | | -- | -- | | -- |
| **Arachidonic acid** | **C20:4 n-6** | 19.665 | | 0.376 | | 13.270 | 0.546 | | 0.000 |
| **Eicosapentaenoic acid)** | **C20:5 n-3** | 0.468 | | 0.038 | | 0.301 | 0.021 | | 0.003 |
| **Erucic acid** | **C22:1 n-9** | 0.395 | | 0.061 | | 0.134 | 0.030 | | 0.003 |
| **Adrenic acid** | **C22:4 n-6** | 1.524 | | 0.085 | | 0.914 | 0.051 | | 0.000 |
| **Docosapentaenoic acid** | **C22:5 n-3** | 3.645 | | 0.113 | | 1.915 | 0.101 | | 0.000 |
| **Docosahexaenoic acid** | **C22:6 n-3** | 0.518 | | 0.021 | | 0.589 | 0.028 | | 0.051 |
| **SFA^1^** |  | 30.124 | | 0.532 | | 30.259 | 0.774 | | 0.884 |
| **MUFA^2^** |  | 11.848 | | 0.307 | | 16.342 | 0.436 | | 0.000 |
| **PUFA3** |  | 41.570 | | 0.789 | | 33.941 | 0.560 | | 0.000 |
| **MUFA/SFA** |  | 0.396 | | 0.013 | | 0.543 | 0.018 | | 0.000 |
| **PUFAn-6^4^** |  | 36.593 | | 0.719 | | 30.829 | 0.497 | | 0.000 |
| **PUFAn-3^5^** |  | 4.978 | | 0.131 | | 3.113 | 0.092 | | 0.000 |
| **∑n-6/∑n-3** |  | 7.402 | | 0.182 | | 9.956 | 0.215 | | 0.000 |
| **C18:1/C18:0** |  | 0.353 | | 0.011 | | 0.492 | 0.016 | | 0.000 |

**Table 6. Fatty-acids composition.** Differences in mean values (%) and S.E.M. for polar lipids in the liver of control (normal diet) and obese sows (obesogenic diet).

^1^SFA = Saturated fatty acids; Includes: C14:0, C16:0, C17:0 and C18:0

^2^MUFA = Monounsaturated fatty acids; Includes: C16:1n-9, C16:1n-7, C17:1, C18:1n-9, C18:1n-7, C20:1n-9 and C22:1n-9.

^3^PUFA = Polyunsaturated fatty acids: Includes: C18:2n-6, C18:3n-3, C20:3n-9, C20:4n-6, C20:5n-3, C22:4n-6, C22:5n-3, C22:6n-3.

^4^Includes: C18.2n-6, C20:4n-6 and C22:4n-6.

^6^Includes: C18:3n-3, C20:5n-3, C22:5n-3 and C22:6n-3.
