# Supplementary material for: Characterization of Ageing- and Diet-Related Swine Models of Sarcopenia and Sarcopenic Obesity
Source: Int J Mol Sci. 2018 Mar 12;19(3):823. doi: 10.3390/ijms19030823 (PMC5877684; doi:10.3390/ijms19030823)
Supplement: Supplementary file 1 [file ijms-19-00823-s001.zip › TABLA 7.docx]

|  |  | | CONTROL | |  | OBESE | |  |  |
| --- | --- | --- | --- | --- | --- | --- | --- | --- | --- |
| **Trivial name** | **Abbreviation** | **Mean** | | **SEM** | | **Mean** | **SEM** | | **P-value** |
| **Myristic acid** | **C14:0** | 0.214 | | 0.012 | | 0.373 | 0.104 | | 0.070 |
| **Palmitic acid** | **C16:0** | 15.770 | | 0.320 | | 19.890 | 0.791 | | 0.000 |
| **cis-7 hexadecenoic acid** | **C16:1 n-9** | 0.325 | | 0.015 | | 0.401 | 0.021 | | 0.005 |
| **Palmitoleic acid** | **C16:1 n-7** | 0.473 | | 0.034 | | 1.059 | 0.369 | | 0.059 |
| **Margaric acid** | **C17:0** | 1.310 | | 0.058 | | 1.080 | 0.126 | | 0.074 |
| **cis-10-Heptadecenoic acid** | **C17:1** | 0.378 | | 0.015 | | 0.430 | 0.020 | | 0.045 |
| **Stearic acid** | **C18:0** | 27.611 | | 0.341 | | 32.643 | 2.273 | | 0.012 |
| **Oleic acid** | **C18:1 n-9** | 11.978 | | 0.317 | | 5.248 | 3.787 | | 0.036 |
| **cis-vaccenic acid** | **C18:1 n-7** | 1.396 | | 0.062 | | 3.225 | 1.907 | | 0.240 |
| **Linoleic acid** | **C18:2 n-6** | 16.650 | | 0.207 | | 17.567 | 1.116 | | 0.331 |
| **Linolenic acid** | **C18:3 n-3** | 0.412 | | 0.014 | | 0.344 | 0.013 | | 0.003 |
| **Eicosenoic acid** | **C20:1 n-9** | 0.544 | | 0.024 | | 0.558 | 0.047 | | 0.771 |
| **Mead acid** | **C20:3n-9** | -- | | -- | | -- | -- | | -- |
| **Arachidonic acid** | **C20:4 n-6** | 16.951 | | 0.226 | | 13.521 | 1.406 | | 0.006 |
| **Eicosapentaenoic acid** | **C20:5 n-3** | 0.345 | | 0.022 | | 0.256 | 0.031 | | 0.025 |
| **Erucic acid** | **C22:1 n-9** | 0.500 | | 0.188 | | 0.059 | 0.006 | | 0.072 |
| **Adrenic acid** | **C22:4 n-6** | 1.716 | | 0.092 | | 1.008 | 0.089 | | 0.000 |
| **Docosapentaenoic acid** | **C22:5 n-3** | 2.912 | | 0.085 | | 1.732 | 0.189 | | 0.000 |
| **Docosahexaenoic acid** | **C22:6 n-3** | 0.516 | | 0.015 | | 0.605 | 0.062 | | 0.103 |
| **SFA^1^** |  | 29.134 | | 0.324 | | 34.096 | 2.257 | | 0.012 |
| **MUFA^2^** |  | 15.594 | | 0.416 | | 10.980 | 4.548 | | 0.218 |
| **PUFA^3^** |  | 39.502 | | 0.240 | | 35.034 | 2.773 | | 0.055 |
| **MUFA/SFA** |  | 0.538 | | 0.019 | | 0.544 | 0.346 | | 0.982 |
| **PUFAn-6^4^** |  | 35.317 | | 0.246 | | 32.096 | 2.518 | | 0.124 |
| **PUFAn-3^5^** |  | 4.185 | | 0.084 | | 2.938 | 0.262 | | 0.000 |
| **∑n-6/∑n-3** |  | 8.497 | | 0.192 | | 11.173 | 0.313 | | 0.000 |
| **C18:1/C18:0** |  | 0.487 | | 0.018 | | 0.496 | 0.350 | | 0.976 |

**Table 7. Fatty-acids composition.** Differences in mean values (%) and S.E.M. for neutral lipids in the liver of control (normal diet) and obese sows (obesogenic diet).

^1^SFA = Saturated fatty acids; Includes: C14:0. C16:0. C17:0 and C18:0

^2^MUFA = Monounsaturated fatty acids; Includes: C16:1n-9. C16:1n-7. C17:1. C18:1n-9. C18:1n-7. C20:1n-9 and C22:1n-9.

^3^PUFA = Polyunsaturated fatty acids: Includes: C18:2n-6. C18:3n-3. C20:3n-9. C20:4n-6. C20:5n-3. C22:4n-6. C22:5n-3. C22:6n-3.

^4^Includes: C18.2n-6. C20:4n-6 and C22:4n-6.

^6^Includes: C18:3n-3. C20:5n-3. C22:5n-3 and C22:6n-3.
